# Repositioning Drugs on Human Influenza A Viruses Based on a Novel Nuclear Norm Minimization Method

**DOI:** 10.3389/fphys.2020.597494

**Published:** 2021-01-18

**Authors:** Hang Liang, Li Zhang, Lina Wang, Man Gao, Xiangfeng Meng, Mengyao Li, Junhui Liu, Wei Li, Fanzheng Meng

**Affiliations:** ^1^Pediatric Department of Respiration II, The First Hospital of Jilin University, Changchun, China; ^2^Norman Bethune Health Science Center, Jilin University, Changchun, China

**Keywords:** influenza A viruses, anti-viral drugs, treatment, drug repositioning, hemagglutinin

## Abstract

Influenza A viruses, especially H3N2 and H1N1 subtypes, are viruses that often spread among humans and cause influenza pandemic. There have been several big influenza pandemics that have caused millions of human deaths in history, and the threat of influenza viruses to public health is still serious nowadays due to the frequent antigenic drift and antigenic shift events. However, only few effective anti-flu drugs have been developed to date. The high development cost, long research and development time, and drug side effects are the major bottlenecks, which could be relieved by drug repositioning. In this study, we proposed a novel antiviral Drug Repositioning method based on minimizing Matrix Nuclear Norm (DRMNN). Specifically, a virus-drug correlation database consisting of 34 viruses and 205 antiviral drugs was first curated from public databases and published literature. Together with drug similarity on chemical structure and virus sequence similarity, we formulated the drug repositioning problem as a low-rank matrix completion problem, which was solved by minimizing the nuclear norm of a matrix with a few regularization terms. DRMNN was compared with three recent association prediction algorithms. The AUC of DRMNN in the global fivefold cross-validation (fivefold CV) is 0.8661, and the AUC in the local leave-one-out cross-validation (LOOCV) is 0.6929. Experiments have shown that DRMNN is better than other algorithms in predicting which drugs are effective against influenza A virus. With H3N2 as an example, 10 drugs most likely to be effective against H3N2 viruses were listed, among which six drugs were reported, in other literature, to have some effect on the viruses. The protein docking experiments between the chemical structure of the prioritized drugs and viral hemagglutinin protein also provided evidence for the potential of the predicted drugs for the treatment of influenza.

## Introduction

Influenza viruses spread quickly and are among the main causes of human death. Influenza is an acute respiratory tract infection caused by influenza viruses that seriously endangers human health. Symptoms include a stuffy nose, cough, sore throat, headache, fever, chills, anorexia, and myalgia. These symptoms are the result of inflammation caused by a viral infection (Eccles, 2005). Type A influenza viruses are major pathogens for humans. Infection with influenza A viruses usually results in mild and self-limiting illness. For some people, they can cause complications such as pneumonia, bronchitis, sinusitis, and ear infections, leading to serious illness and even death (CDC, 2009). Influenza complications are often associated with secondary bacterial infections, which may be due to the virus inducing a series of changes in the host lung epithelial cells, making them easy to adhere and invade, leading to changes in the immune response (Mccullers, 2006, 2014; Jamieson et al., 2013). Influenza A viruses are evolving very fast, which allows them to regularly produce new strains of human immunodeficiency, leading to periodic pandemics (Taubenberger and Kash, 2010). Among the known 16 hemagglutinin (HA) subtypes and nine neuraminidase (NA) subtypes of influenza A viruses, only H3N2 subtypes and H1N1 subtypes are currently spreading among the population (Webster et al., 1992).

Prevention and treatment of influenza A viruses usually use vaccines or anti-flu chemical drugs. However, the effectiveness of the vaccine is based on the similarity of the vaccine strain to the influenza virus strain that is circulating (Tosh and Poland, 2008). Influenza viruses continue to mutate, and conventional vaccines may not easily prevent or treat influenza outbreaks caused by new viruses. Therefore, the research of anti-influenza chemical drugs is of great significance (Glezen, 2006). Two types of drugs commonly used to prevent or treat influenza A viruses are amantadine and neuraminidase inhibitors (NAIs). Studies have shown that the effectiveness of amantadine is limited by the high prevalence of influenza A virus (H3N2) with the S31N mutation in M2 (Barr et al., 2007; Saito et al., 2007). In 2008, the H1N1 subtype with the H274Y mutation in NA appeared, which raised concerns about the use of oseltamivir (Hauge et al., 2009; Hurt et al., 2009a). On the other hand, the incidence of zanamivir-resistant viruses is low. Chemiluminescence NAI analysis confirmed that the H3N2 subtype with the D151A/V mutation in NA reduces the sensitivity of zanamivir (Sheu et al., 2008). It has been reported that an H1N1 subtype isolate with a new Q136K mutation in NA that is resistant to zanamivir has been isolated in Oceania and Southeast Asia (Hurt et al., 2009b). Burch et al. (2009) commissioned by the National Institute of health and clinical optimization, searched the database of studies on the use of neuraminidase inhibitors in the treatment of seasonal influenza. They presented the results to healthy adults (i.e., adults without known comorbidities) and people at risk for influenza-related complications (Burch et al., 2009). Rohloff et al. (1998) prepared GS-4104, an anti-influenza drug of 3,3-Diaryloxidoles, with a high yield (62–99%), through an isobutyl or substituent reaction.

Nevertheless, the development of new drugs for the prevention and treatment of influenza A viruses is a long process with a high cost. Therefore, repeated use of drugs is a strategy to find specific drugs for the treatment of influenza A viruses among existing drugs. Compared with developing new drugs, it can greatly shorten the time and reduce the cost. However, blindly repeating the use of drugs and randomized clinical trials is risky, and there is still the problem that they are time-consuming and costly. At present, some calculation methods provide new testable hypotheses for the repositioning of systemic drugs (Cheng et al., 2016; Santos et al., 2017). Therefore, more computational methods for drug screening are urgently needed to find drugs that may have therapeutic effects against Influenza A viruses and thereby solve these time-consuming and costly problems.

In this study, we developed a matrix decomposition-based antiviral drug reuse method to predict the efficacity of drugs for the treatment of influenza A virus (H3N2), and the method mainly includes the following four steps: (1) collect and download data about viruses and drugs from the literature; (2) calculate a similar chemical structure of the drugs and similar genetic sequence of the virus; (3) establish a heterogeneous drug-virus network based on the virus and drug-related data, the drug similarity network, and the virus similarity network; (4) use the nuclear norm minimization method to obtain the drug most likely to have a therapeutic effect on the virus. Finally, the experiment evaluated the performance of this method through fivefold CV, and the results showed that DRMNN achieved an average AUC value of 0.8661.

## Materials and Methods

### Human Virus and Drug Interaction Associations

In order to construct a human virus–drug interaction network, we used text mining technology to study a large number of previous documents and screened a drug database, and we finally found 408 confirmed human virus-drug interaction associations, including 34 viruses and 205 drugs. The adjacency matrix variable of the virus-drug interaction network was defined as *A*. If the drug *d*(*i*) has an effect on the virus *v*(*j*), then *A*(*ij*) is equal to 1, otherwise it is 0. That is:

$$A\,\left(i\,j\right)=\begin{array}{cc} 1, & i\,f\,d\,r\,u\,g\,d\,\left(i\right)\,h\,a\,s\,a\,n\,e\,f\,f\,e\,c\,t\,o\,n\,t\,h\,e\,v\,i\,r\,u\,s\,v\,\left(j\right)\\ 0, & o\,t\,h\,e\,r\,w\,i\,s\,e \end{array}$$

### Chemical Structure Similarity of Drugs

The drug discovery process is characterized by a long cycle, high investment, and high risk. In order to shorten the drug development cycle and control the risk and cost of the drug development process, computer-aided drug design (CADD) has become an important tool for new drug development and drug screening. Molecular similarity calculation is widely used in the CADD field. Molecular shape similarity is usually based on the Tanimoto Coefficient (TC). The MACCS fingerprint in Openbabel V2.3.1 software was used to calculate the molecular fingerprint similarity between two drugs, represented by TC. Drugs’ chemical structure information was downloaded from the DrugBank database. If the MACCS fragment bit strings of two drug molecules *d*(*i*) and *d*(*j*) were *m*(*i*) and *m*(*j*), respectively, *a* was set as the fingerprints of the two drugs. The similarity between drugs *d*(*i*) and *d*(*j*) was defined as:

(1) $$D\,S\,\left(d\,\left(i\right),d\,\left(j\right)\right)=T\,C=\frac{a}{m\,\left(i\right)+m\,\left(j\right)-a}$$

The *TC* value ranges from zero (no common bits) to one (all bits are the same), and it can be widely used in various drug development and repositioning processes. The chemical structure similarity matrix of drugs is represented by *DS*. Finally, the calculated drug similarity constitutes a medical chemical structure similarity network.

### Viral Similarity

Our understanding of any virus often starts from its sequence. With the development of gene sequencing technology, a lot of multiple sequence comparison software has also emerged. MAFFT is a multi-sequence alignment program (Katoh et al., 2005) that provides a series of alignment methods with the advantages of fast alignment and high accuracy. Therefore, we used MAFFT to calculate the sequence similarity between viruses to express the similarity between viruses. Then, we constructed a viral similarity network and used *VS* to represent the viral similarity matrix.

### Human Virus-Drug Interactome Network

We constructed a human virus-drug interactome network by using human virus and drug interaction associations, a network of chemical structure similarity of drugs, and a virus similarity network. Then, the heterogeneous human virus-drug interactome network was treated as a bipartite graph *G*(*V*,*D*,*E*), where *V* represented human viruses, *D* represented drugs, and *E* was the edges connecting human viruses and drugs. Therefore, the adjacency matrix of the heterogeneous drug-virus network matrix can be defined as:

(2) $$B=\left[\begin{array}{cc} D\,S & A^{T}\\ A & V\,S \end{array}\right]$$

where *A*^*T*^ is the transposition of *A*.

### DRMNN

**FIGURE 1:**
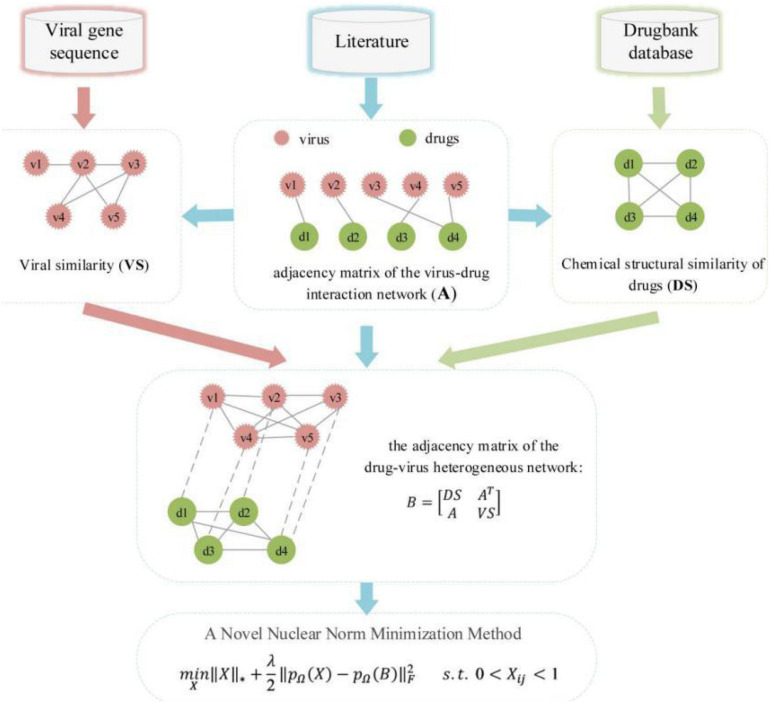
The workflow of DRMNN.

An overview of DRMNN was shown in Figure 1. The nuclear norm is the sum of the singular values of the matrix, which is used to constrain the low rank of the matrix. For sparse data, the matrix has a low rank and contains a lot of redundant information, which can be used to recover data and extract features. The nuclear norm has been widely used in various fields and has achieved good results (Yang et al., 2019). Generally, when a matrix has a low rank, the kernel norm minimization problem can be expressed as:

(3) $$m\,i\,n\,X\,{||X||}_{*}$$

where ||*X*||_*_ represents the kernel norm of *X*, which is defined as the sum of all singular values of *X*. The kernel norm minimization model is a convex optimization problem.

In order to predict the drug-virus association, the elements in the drug similarity matrix *DS* and the virus similarity matrix *VS* are in the interval [0, 1]. The elements in the correlation matrix *A* are 0 or 1. The predicted value of the unknown entry is expected to be in the range of [0, 1], where a predicted value close to 1 suggests that it may be indicative of an association and vice versa. However, in the above matrix completion model (2), the entries in the completed matrix can be any real values in (−, +). However, it has no practical significance for values greater than 1 and less than 0. Therefore, it is important to add a constraint to the matrix completion model to ensure that the missing elements that are not found are in the interval [0, 1]. In addition, because there may be a lot of “noise” data in the drug and virus data, the drug relocation model should tolerate the potential noise as much as possible. The noise-tolerant matrix completion model is:

(4) $$m\,i\,n\,X\,{||X||}_{*}\,s.t.{||p_{\Omega }\,\left(X\right)-p_{\Omega }\,\left(B\right)||}_{F}\leq \varepsilon$$

where ε is the measurement noise level, Ω is a set of index pairs (*i*,*j*) containing all known entries in *B*, and *p*Ωis the projection operator on Ω.

(5) $${\left(p_{\Omega }\,\left(X\right)\right)}_{i\,j}=\left\{ \begin{array}{cc} X_{i\,j}, & \left(i,j\right)\in \Omega \\ 0, & o\,t\,h\,e\,r\,w\,i\,s\,e \end{array}\right.$$

However, there are a number of difficulties involved in solving this model with its inequality constraints, for example, how to choose the appropriate model parameters and how to choose an effective solution algorithm. Therefore, we usually replace the inequality constraint model with a regularized model. The introduction of soft regularization can tolerate unknown noise and also make the solution much more convenient to arrive at. Then the model can be rewritten as the following:

(6) $$\underset{X}{m\,i\,n}{||X||}_{*}+\frac{\lambda }{2}\,{||p_{\Omega }\,\left(X\right)-p_{\Omega }\,\left(B\right)||}^{2_{F}}\,s.t\,. 0<X_{i\,j}<1$$

where ||⋅||_*F*_ denotes the Frobenius norm and λ is the parameter that balances the nuclear specification and the error term. To solve the optimization problem in Eq. (5), we chose the more classic alternating direction multiplier method (ADMM) (Gu et al., 2016). It is worth noting that our objective function is convex. With the introduction of the auxiliary matrix *H*, the ADMM framework can be optimized in the following equivalent form.

(7) $$\underset{X}{m\,i\,n}{||X||}_{*}+\frac{\lambda }{2}\,{||p_{\Omega }\,\left(X\right)-p_{\Omega }\,\left(B\right)||}^{2_{F}}\,s.t.X=H,0<H_{i\,j}<1$$

Therefore, the enhanced Lagrange function becomes the following:

(8) $$ℒ\,\left(H,X,Y,\lambda ,\mu \right)={||X||}_{*}+\frac{\lambda }{2}\,{||p_{\Omega }\,\left(X\right)-p_{\Omega }\,\left(B\right)||}^{2_{F}}+T_{\gamma }\,\left(Y^{T}\,\left(X-H\right)\right)+\frac{\mu }{2}\,{||X-H||}^{2_{F}}$$

where *Y* is the Lagrange multiplier and μ > 0 is the penalty parameter. The solution process of DRMNN belongs to an iterative solution. Therefore, when we iterate *k* times, we need to calculate the value of iterations *H*_*k+1*_, *Y*_*k+1*_, and *X*_*k+1*_ according to the result of the *k*^*th*^ iteration.

Update: Repeat the following steps until there is convergence or a predetermined number of iterations.

Fix *X_k* and *Y_k* and calculate a matrix *H*_*k+1*_ to minimize Eq. (7).

(9) $$H_{k+1}=a\,r\,g\,m\,i\,n_{0\leq H\leq 1}\,ℒ\,\left(H,X_{k},Y_{k},\lambda ,\mu \right)=a\,r\,g\,m\,i\,n_{0\leq H\leq 1}\,\frac{\lambda }{2}\,{||p_{\Omega }\,\left(X\right)-p_{\Omega }\,\left(B\right)||}^{2_{F}}+T_{\gamma }\left(Y^{T(X_{k-H}}\right))+\frac{\mu }{2}||X{}_{k-H}||{}^{2_{F}}$$

Here, *H*^∗^ is the optimal solution of *argmin*_0≤*H*≤1_ℒ(*H*,X_k_,Y_k_,λ,μ), if and only if

(10) $$\lambda \,p_{\Omega }^{*}\,\left(p_{\Omega }\,\left(H^{*}\right)-p_{\Omega }\,\left(B\right)\right)-Y_{k}-\mu \,\left(X_{k-H^{*}}\right)=0$$

holds, where $p_{\Omega }^{*}$ represents the adjoint operator of *p*Ω. Then, the closed solution becomes:

$$H^{*}={\left(\alpha +\frac{\lambda }{\mu }\,p_{\Omega }^{*}\,p_{\Omega }\right)}^{-1}\,\left(\frac{1}{\mu }\,Y_{k}+\frac{\lambda }{\mu }\,p^{{*}_{\Omega }}\,p_{\Omega }\,\left(B\right)+X_{k}\right)=\left(\alpha -\frac{\lambda }{\mu }\,p_{\Omega }^{*}\,p_{\Omega }\right)\,\left(\frac{1}{\mu }\,Y_{k}+\frac{\lambda }{\mu }\,p^{{*}_{\Omega }}\,p_{\Omega }\,\left(B\right)+X_{k}\right)$$

(11) $$=\left(\frac{1}{\mu }\,Y_{k}+\frac{\lambda }{\mu }\,p_{\Omega }\,\left(B\right)+X_{k}\right)-\frac{\lambda }{\mu +\lambda }\,\left(\frac{1}{\mu }\,Y_{k}+\frac{\lambda }{\mu }\,p_{\Omega }\,\left(B\right)+X_{k}\right)$$

where α is the identity operator. ${\left(\alpha +\frac{\lambda }{\mu }\,p_{\Omega }^{*}\,p_{\Omega }\right)}^{-1}$ denotes the inverse operator of $\left(\alpha +\frac{\lambda }{\mu }\,p_{\Omega }^{*}\,p_{\Omega }\right)$, and it is equal to $\left(\alpha -\frac{\lambda }{\mu }\,p_{\Omega }^{*}\,p_{\Omega }\right)$. It’s worth noting that $p_{\Omega }^{*}\,p_{\Omega }\,p_{\Omega }$. Considering the interval [0,1] constraint, we limit the range of the elements of *H*_*k+1*_ to [0, 1] such that

(12) $${\left(H_{k+1}\right)}_{ij}=\{\begin{array}{cc} 1, & H_{ij}^{*}>1\\ H_{ij}^{*}, & 0<H_{ij}^{*}<1\\ 0, & H_{ij}^{*}<0 \end{array}$$

Fix *H*_*k+1*_ and *Y_k* and calculate a matrix *X*_*k+1*_ to minimize Eq. (7).

(13) Xk+1=argm⁢i⁢n0≤H≤1ℒ(H,k+1X,Yk,λ,μ)=a⁢r⁢g⁢m⁢i⁢n0≤H≤1⁢||X||*+μ2⁢||X-(Hk+1-1μ⁢Yk)||2F=ϑ1θ⁢(Hk+1-1μ⁢Yk)

where *ϑ*_τ_(*X*) is the singular value shrinkage operator which is defined as:

(14) $$\vartheta_{\tau }\,\left(X\right)=\int_{i=1}^{\theta_{i}\geq \tau }\left(\theta_{i}-\tau \right)\,\beta_{i}\,\gamma^{T_{i}}$$

where β_*i*_ and γ_*i*_ are the left and right singular vectors corresponding to θ_*i*_, respectively. The θ_*i*_ are the singular values of *X*, which are greater than τ.

Fix *H*_*k+1*_ and *X*_*k+1*_ and calculate a matrix *Y*_*k+1*_.

(15) $$Y_{k+1}=Y_{k}+\kappa \,\mu \,\left(X_{k+1}-H_{k+1}\right)$$

where κ is the learning rate which is set to 1 in this study for simplicity. Iterate according to the above iteration rules until convergence, and finally, we obtain the matrix *H_k* after convergence. Therefore, the final prediction matrix *A*^∗^ for potential association between drugs and viruses is

(16) A*←[D⁢S*A*TA*V⁢S*]←Hk

## Results

### Indicators of Performance Evaluation

For a binary classification problem, the samples are generally divided into two types: positive samples and negative samples. In dichotomies, therefore, there are usually the following four situations:

TP: True Positives, which means the number from the sample itself that are positive and are predicted to be positive;
FP: False Positives, which means the number of samples that are negative and ultimately predicted to be positive;
TN: True Negatives, which indicates the number of negatives from the sample itself that are also predicted to be negative;
FN: False Negatives, which indicates the number of positives that the sample itself ultimately predicted to be negative.

The commonly used evaluation indicators of classification models are: precision, specificity, and sensitivity. Their calculation formula is as follows:

$$A\,C\,C=\frac{T\,P+T\,N}{T\,P+F\,P+T\,N+F\,N}$$

$$P\,r\,e\,c\,i\,s\,i\,o\,n=\frac{T\,P}{T\,P+F\,P}$$

$$S\,p\,e\,c\,i\,f\,i\,c\,i\,t\,y\,\left(1-F\,P\,R\right)=\frac{T\,N}{T\,N+F\,P}$$

$$Sensitivity\left(TPR=Recall\right)=\frac{T\,P}{T\,P+F\,N}$$

The performance evaluation indicators we usually adopt are the ROC curve and area under the ROC curve (AUC value), as well as the PR curve and area under the PR curve (AUPR value). The full name of the ROC is Receiver Operating Characteristic, its abscissa is the false positive rate (FPR), and its ordinate is the true positive rate (TPR). Among them, the false positive rate is the proportion of all negative samples that the classifier incorrectly predicts as positive, also known as 1-specificity. Similarly, the true positive rate refers to the proportion of positive samples correctly identified by the classifier out of all positive samples, which is also called sensitivity. Then we draw the ROC curve based on the TPR and FPR. Calculate the area under the ROC curve and perform a numerical evaluation of the model’s performance. The area under the ROC curve is defined as AUC (Area Under Curve). AUC = 0.5 means completely random prediction, and AUC = 1 means a completely accurate prediction. Obviously, the area is less than 1, but the larger the AUC, the better the performance of the classifier. On the other hand, the PR curve is actually made by using precision and recall as variables, where recall is the *x*-coordinate and precision is the *y*-coordinate. AUPR represents the area under the PR curve. The closer AUPR is to 1, the better the prediction’s performance will be.

### Performance in Predicting Virus-Drug Association

In DRMNN, there are two parameters λ and μ that need to be determined. For λ and μ, they are determined from {0.1, 1, 10, 100}, respectively. We performed fivefold CV on the training data set to determine the parameters and found that when λ = 1 and μ = 10, DRMNN performs best. The AUC results are shown in Table 1.

**TABLE 1:** The AUC values using different λ and μ values in fivefold CV on the dataset.

λ\μ	0.1	1	10	100
0.1	0.7044	0.6992	0.8087	0.8517
1	0.8116	0.8123	**0.8661**	0.8378
10	0.7919	0.7983	0.8589	0.8318
100	0.7823	0.8006	0.8536	0.8276

*The bold value indicates that AUC.*

In order to evaluate the prediction performance of DRMNN, we applied DRMNN to the known human virus and drug interaction associations *A* and used the fivefold CV to evaluate its performance. The specific process was as follows: all known human virus and drug interaction associations were randomly divided into five uncrossed sites with equal size. We used one of the parts as a test sample for prediction and the other four parts of the sample as training data to build a predictive model. This process was repeated five times and ended when all samples were predicted once. The results showed that the AUC value was 0.8661. The AUPR value was 0.4442.

At present, there are few algorithms for predicting which drugs will effectively treat influenza A viruses by constructing a network of viruses and drugs. Therefore, in this study, we compared network association prediction algorithms in other fields to explore the performance of DRMNN in predicting drugs that can treat influenza A viruses. NCP was first proposed by Gu et al. (2016) to predict miRNA-disease association. Zou et al. (2018) used this method to predict the association between microorganisms and diseases and achieved good results. NCP is a method based on a general nonparametric network, which belongs to the category of unsupervised learning. Its characteristic is that no negative samples are required. The Random Walk with Restart (RWR) algorithm has advantages. It is not only used for the correlation prediction of binary networks, but also for link prediction of various heterogeneous networks, and in various network correlation predictions, the RWR algorithm shows good predictive performance (Chen et al., 2012). The inductive matrix completion (IMC) algorithm and collaborative matrix factorization algorithm (CMF) (Xu et al., 2020) were more commonly used in prediction problems. The IMC algorithm was originally used to predict the association between drugs and targets, and was finally applied by Chen et al. in miRNA-disease association networks, which also showed good performance (Chen et al., 2018). In the analysis of all the above methods, RWR and IMC contain parameters that need to be fine-tuned. For all parameters, we select the best parameters by using a global fivefold CV.

**FIGURE 2:**
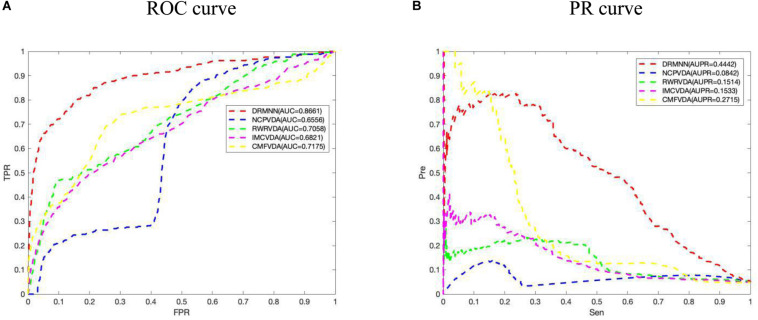
Comparison of predictive performance of DRMNN, NCP, RWR, CMF, and IMC. **(A)** ROC curve and AUC value based on the global fivefold CV. **(B)** PR curve and AUPR values based on global fivefold CV.

We applied DRMNN, NCP, RWR, IMC, and CMF to the 341 associated data between 34 viruses and 205 drugs that were considered. Under the global fivefold CV, the final AUC values of them were 0.8661, 0.6556, 0.7058, 0.6821, and 0.7175, respectively. The ROC curve is shown in Figure 2A, indicating that DRMNN showed the best performance in predicting the association between viruses and drugs. We also draw the PR curve in Figure 2B. The AUPR of DRMNN, NCP, RWR, CMF, and IMC were 0.4442, 0.0842, 0.1514, 0.1533, and 0.2715, respectively. This once again proves that DRMNN performs best in predicting the treatment of influenza A virus.

**FIGURE 3:**
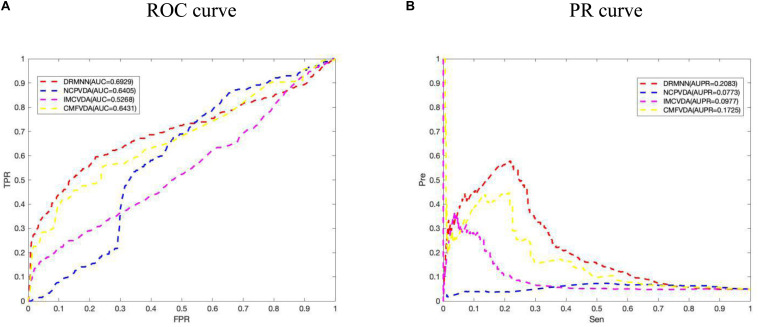
Comparison of predictive performance of DRMNN, NCP, CMF, and IMC. **(A)** ROC curve and AUC value based on the local LOOCV. **(B)** PR curve and AUPRs value based on the local LOOCV.

In addition, we also carried out a local LOOCV. In particular, for each virus *v*(*i*), we removed all known drugs associated with virus *v*(*i*), and used the remaining data to build prediction models. But RWR cannot predict new virus-related drugs, so RWR was removed, and only a few other algorithms were compared. The ROC curve and PR curve are shown in Figure 3. The results show that the AUC value and the AUPR value of DRMNN are 0.6929 and 0.2083, respectively, which are much higher than with the other three algorithms. The local LOOCV results also show that DRMNN performed well in predicting potential therapeutic agents for new viruses.

## Case Study

### Identification of Potential Drugs Against the Influenza A Virus (H3N2)

Accurately determining the drugs to use to treat influenza A virus (H3N2) is the primary task of this study. Through the construction of the viral drug network, we used the DRMNN algorithm to select drugs that may be used to treat influenza A virus (H3N2) and help medical staff choose drugs from a computational perspective. During the construction of the network, the influenza A virus (H3N2) had no association with any drugs. DRMNN was used to predict the probability scores of candidate drugs, and the top 10 drugs for the treatment of influenza A virus (H3N2) are shown in Table 2. Nitrazoxanide (ranked 2) can be used to treat *Cryptosporidium parvum* and Giardia infections in children and adults, and it has been licensed in the United States; it is a safe, oral, bioavailable anti-infective drug (White, 2004). In addition to being used to treat protozoan and bacterial infections, thiazoles are also used as a class of broad-spectrum antiviral drugs (Rossignol et al., 2006, 2009a,b; Korba et al., 2008; Elazar et al., 2009; La Frazia et al., 2013). These molecules selectively block the maturation of the viral hemagglutinin through a stage before the resistance to endoglycosidase H digestion and disrupt the intracellular transport and insertion of the HA into the host cell plasma membrane for the correct assembly of the virus and its removal from the host cell to fight off the virus. Studies have found that Nitrazoxanide is effective against influenza A virus (H3N2), which contains the M2 blocker resistance marker S31N (Sleeman et al., 2014). Chloroquine (ranked 3) is a 9-aminoquinolone that can be used to fight malaria and that has biochemical properties that can be used to inhibit virus replication. The report pointed out that chloroquine can inhibit the replication of influenza A virus *in vitro*, and the IC50s of chloroquine to influenza A virus H3N2 are lower than the plasma concentration reached during acute malaria treatment (Ooi et al., 2006). Umifenovir (ranked 8) is licensed in Russia and is widely used for the prevention or treatment of influenza. Leneva et al. (2019) found that Umifenovir effectively inhibited the replication of antigen-dominant human type A influenza virus using MDCK cell-based enzyme linked immunoadsorption assay (ELISA), and none of the viruses isolated before and during umifenovir treatment showed reduced sensitivity to neuraminidase (NA) inhibitors, suggesting that umifenovir is effective in treating influenza A virus.

**TABLE 2:** Top 10 possible drugs against influenza A virus (H3N2) predicted by DRMMN.

Virus	Rank	Drug name	Evidence
Influenza A virus (H3N2)	1	Ribavirin	Unconfirmed
	2	Nitazoxanide	Confirmed
	3	Chloroquine	Confirmed
	4	Favipiravir	Unconfirmed
	5	Camostat	Unconfirmed
	6	Mizoribine	Confirmed
	7	Niclosamide	Confirmed
	8	Umifenovir	Confirmed
	9	Mycophenolic acid	Unconfirmed
	10	Amantadine	Confirmed

### Molecular Docking

**FIGURE 4:**
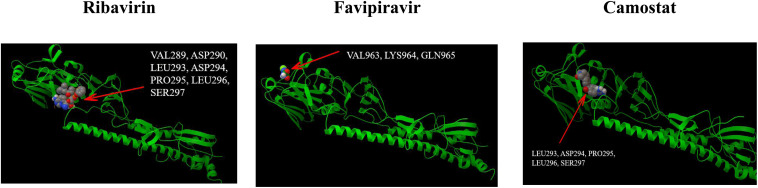
The binding mode between the four drugs docked with molecules and the receptor HEMAGGLUTININ (PDB ID: 2VIU).

Molecular docking research has become an economic and modern trend in drug development. It can be used to design known ligands for specific active sites of macromolecules, and it is a method that provides valuable information. The technology-based ligand-protein interaction reveals the possibility of pre-synthesis. In our study, the computer chemistry research of the top five drugs predicted by DRMNN was being blindly connected in online and offline modes. The Autodock 4.2 package^1^ was used for offline docking. The X-ray crystal structure of the protein was searched from the RCSB protein database^2^. The PDB ID is a 2VIU macromolecule, which is the receptor binding the domain of influenza A virus (H3N2) complexed with its receptor Hemagglutinin. We used MGL Tools 1.5.6 and Autodock Tool (ADT) to prepare all proteins and ligands. ADT was used to calculate the binding free energy and inhibition constant of the optimal docking complex of the above proteins. Figure 4 showed the interaction of three unproven drugs predicted by DRMNN with important residues on their receptor Hemagglutinin. The negative combination free energy further indicates the stability of the complex (Table 3). This evidence all showed that the drugs predicted by DRMNN are effective in suppressing influenza A viruses.

**TABLE 3:** The binding affinity of the unconfirmed drugs predicted by DRMNN to the target PDB ID: 2VIU.

*Drug name*	Ribavirin	Favipiravir	Camostat
*Free energy of binding (kcal/mol)*	−7.4	−5.2	−7.5

## Discussion

Influenza A viruses have always been among the most important viruses harmful to human health. They can cause acute respiratory infection that is harmful to human health and is one of the main causes of death. To prevent and treat influenza viruses, vaccines or anti-influenza chemicals are usually used. However, traditional vaccines may not easily prevent and treat influenza outbreaks caused by new viruses, while the development of new drugs will require longer time and higher economic costs. Therefore, strategies to find effective drugs among existing drugs can greatly reduce time and cost. In this paper, we propose a method of reuse of antiviral drugs based on the minimum nuclear specification. The method mainly uses data collected from the literature on viruses and drugs, combines the similarity of drug chemical molecules with the similarity of virus gene sequence, and uses DRMNN to obtain the drug most likely to treat influenza A virus (H3N2). After global fivefold CV, DRMNN showed better performance than other methods in determining the treatment of influenza A virus (H3N2). Finally, we obtained the top 10 potential drugs, of which six have been shown to be effective against influenza A virus (H3N2). This method saves the experimental cost and time and provides a powerful reference for preventing and treating influenza A virus.

Although DRMNN has been shown to offer many potential drugs for influenza A virus (H3N2) that may have therapeutic effects, some limitations remain in this study. The DRMNN database contains 13,563 drug entries, and there are thousands of antivirals for broad-spectrum drugs and thousands of viruses for NCBI. Due to the limited amount of data, there are still some biases in the potential drugs we obtain. Therefore, determining how to select effective data to establish greater data integration is an important goal for future research.

Finally, we focused our analyses on influenza in this study. However, it is clear that our method could also be applied to other viruses, for example SARS-CoV-2. The outbreak of SARS-CoV-2 has become a serious pandemic and has caused the deaths of hundreds of thousands of people. Currently, there is no confirmed drug effective against this virus. In the future, we will check the drugs predicted by our method for use against this virus and validate their efficacy through both protein docking and wet-lab experiments.

## Data Availability Statement

The original contributions presented in the study are included in the article/supplementary material, further inquiries can be directed to the corresponding author/s.

## Author Contributions

FM conceived, designed, and managed the study. HL and LZ performed the experiments and drafted the manuscript. XM, ML, JL, and WL provided computational support and technical assistance. XM, MG, and LW reviewed the manuscript. All authors approved the final manuscript.

## Conflict of Interest

The authors declare that the research was conducted in the absence of any commercial or financial relationships that could be construed as a potential conflict of interest.

## Footnotes

1 http://autodock.scripps.edu

2 www.rscb.org
